# The role of increased post-impact ball speed on plantar pressure during topspin and slice longline forehand groundstrokes in female tennis players

**DOI:** 10.1186/s13104-023-06614-6

**Published:** 2023-11-13

**Authors:** Johanna Lambrich, Thomas Muehlbauer

**Affiliations:** Division of Movement and Training Sciences, Biomechanics of Sport, University of Duisburg- Essen, Gladbecker Str. 182, 45141 Essen, Germany

**Keywords:** Racket sport, Stroke type, Lower extremity, Pressure-detecting insoles, Plantar loading, Force, Biomechanics

## Abstract

**Objective:**

Performing groundstrokes is a fundamental skill for tennis players. However, little is known about changes in plantar pressure when post-impact ball speed is increased during topspin and slice groundstrokes. The objective of the present study was to examine how elite (International Tennis Number ≤ 2) female tennis players (*N* = 15, mean age: 22.7 ± 7.8 years) change their plantar pressure in the dominant (equals the stroke arm) and non-dominant foot when executing topspin and slice longline forehand groundstrokes in order to increase post-impact ball speed (i.e., 80 km/h, 90 km/h, 100 km/h, *v*_max_).

**Results:**

The repeated measures analysis of variance revealed a significant ball speed × foot dominance interaction. Post-hoc analyses showed larger mean forces during topspin compared to slice groundstrokes for the dominant foot (*p* ≤ .026, *d* ≥ 0.34) but lower values for the non-dominant foot (*p* ≤ .050, *d* ≥ 0.28). Further, with increasing post-impact ball speed, increases in mean forces in both feet during topspin could be observed but only in the dominant foot during slice groundstrokes. Varying mean forces depending on the stroke type and foot dominance imply that specific physical exercises related to these two factors are necessary to optimise plantar pressure distribution.

## Introduction

Tactics in modern tennis were substantially changed by the adoption of topspin groundstrokes [1, 2]. In the past, flat groundstrokes with the forehand and backhand were used almost exclusively to increase the chance for a clean contact point. In contrast, groundstrokes with varying degrees of spin are applied in modern tennis. Here primarily topspin, but also slice (backspin) strokes are used [3]. Due to the added spin, the flight and impact characteristics of the ball change [4]. More precisely, the topspin groundstroke allows higher ball speeds, which reduces the probability of faults (i.e., net or out) [5]. The slice groundstroke on the other hand is primarily used in drop or approach shots [1, 4], where the altered flight trajectory results in a high and slow bounce of the ball. The main kinematic difference between topspin and slice groundstrokes was found in the direction of the racket-head path. While the topspin groundstroke involves a forward-upward racket trajectory, the slice stroke requires a forward-downward motion [1, 2, 4]. These movement discrepancies result in different post-impact ball speeds despite the same racket head speed [1].

Notwithstanding the knowledge that has been gained from the previously mentioned kinematic studies [1, 2], kinetic analyses of topspin and slice groundstroke at increasing post-impact ball speeds are still lacking. Therefore, the aim of this study was to investigate the role of increased post-impact ball speed on plantar pressure during topspin and slice longline forehand groundstrokes. Based on previous studies [6, 7] discussing the influence of post-impact ball speed, we hypothesised that an increase in post-impact ball speed will result in plantar pressure changes in the dominant foot (i.e., increase) versus non-dominant foot (i.e., decrease), irrespective of stroke type. Derived from a kinematic study [1], we further assumed that pressure values will be higher during topspin compared to slice longline groundstrokes, regardless of post-impact ball speed level. The particular relevance of this study is to derive specific recommendations for exercises to obtain optimal plantar pressure distribution depending on the specific stroke type [8].

## Methods

### Participants

Power analysis (G*Power, v3.1.9.7) showed that for a repeated measures analysis of variance (ANOVA) a minimum of 13 players would be required to detect significant differences (assuming 1-*β* = 80%, *α* = 0.05 Cohen’s *f* = 0.25). Twelve right-handed and three left-handed elite female tennis players (mean age [range]: 22.7 ± 7.8 [14–41) years; height: 171.6 ± 6.7 [161–183] cm; mass: 65.6 ± 7.3 kg [52–74]; training experience: 16.3 ± 7.2 [7–32] years; tennis training volume: 10.3 ± 5.1 [1–17] hours/week; athletic training volume: 4.5 ± 3.4 [5-10] hours/week) were recruited from local tennis clubs in the Rhine-Ruhr region (Germany). Only female players with an International Tennis Number (ITN) ≤ 2 that were free from musculoskeletal, neurological, or orthopaedic disorder within the preceding three months were eligible for this study.

### Testing procedures

**Fig. 1:**
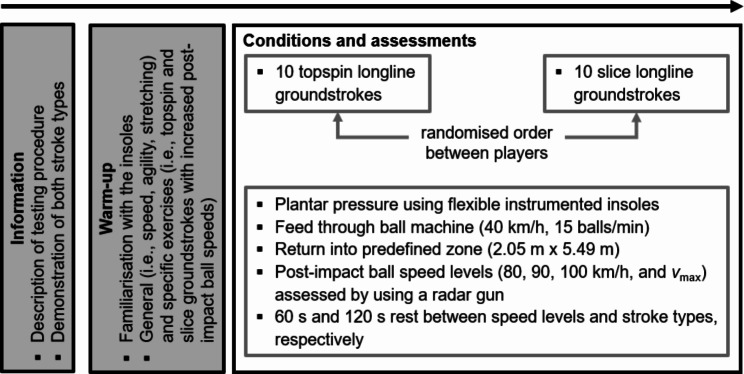
Schematic diagram of the applied testing procedure

The study was performed on an indoor hardcourt in May 2023 using a single-group repeated-measures design (Fig. 1). Upon entering the court, the players received information about the testing procedure and viewed a demonstration of both stroke types. Thereafter, they performed a warm-up of ten minutes during which they got accustomed to the pressure-detecting insoles. The warm-up included speed, agility, and stretching exercises as well as the practice of groundstrokes with their own racket for each stroke type with increased post-impact ball speed. Subsequently, the players were asked to perform data-acquisition groundstrokes per stroke type with increased post-impact ball speed (i.e., 80 km/h, 90 km/h, 100 km/h, *v*_max_) until ten strokes per style and speed level reached a predefined target zone (i.e., 2.05 m x 5.49 m). For each player, new balls were projected from a ball machine (Slinger Bag, Slinger, Windsor Mill, MD, USA) at 40 km/h (feed: 15 balls/min). The order of the two stroke types was randomised between players. Players rested 60 and 120 s between speed levels and stroke types respectively. Post-impact ball speed was assessed using a “Stalker Pro” radar gun (Applied Concepts Inc., Richardson, TX, USA) that was positioned behind the players and the achieved speed was communicated verbally after each stroke.

### Assessment and analysis of plantar pressure data

Plantar pressure distribution (sampling frequency: 200 Hz) was collected using flexible instrumented insoles (GP MobilData WiFi, GeBioM mbH, Münster, Germany). Data were recorded for the dominant (equals stroke arm) and non-dominant foot separately and sent to a laptop wirelessly. Validity as well as reliability of the pressure-detecting insoles for static (standing) and dynamic (walking, running, jumping) movements has been shown in previous research [9]. Each player wore insoles in accordance with her shoe size. Data analysis was performed with MATLAB software version R2022b (The MathWorks Inc., Natick, MA, USA) and included the calculation of the following normalised parameters: maximal force (N/kg), mean force (N/kg), and force-time integral (Ns/kg).

### Statistical analysis

Prior to the conduction of parametric analyses, normal distribution (Shapiro–Wilk Test) and variance homogeneity (Mauchly Test) were checked and confirmed. Data were analysed with a 4 (ball speed: 80 km/h, 90 km/h, 100 km/h, *v*_max_) × 2 (stroke type: topspin, slice) × 2 (foot dominance: dominant, non-dominant) repeated measures ANOVA using JASP version 0.16.4.0 (Amsterdam, The Netherlands). If a significant interaction occurred, Bonferroni-adjusted post-hoc analyses were performed. Further and to analyse groups of related dependent variables that represent different measurements of the same attribute, General Linear Model (GLM) contrasts (type: simple to test for differences among the levels of a factor) were calculated to investigate changes in plantar pressure parameters with increased post-impact ball speed, where 80 km/h served as reference category. Descriptive data are reported as group means ± standard deviations. Normality (Shapiro–Wilk Test) and homogeneity of variance/sphericity (Mauchly Test) were checked and met prior to the application of inference statistics. Partial eta-squared (*η*_p_^2^) was calculated and reported as small (0.02 ≤ *η*_p_^2^ ≤ 0.12), medium (0.13 ≤ *η*_p_^2^ ≤ 0.25), or large (*η*_p_^2^ ≥ 0.26) for the ANOVA and Cohen’s *d* was determined and interpreted as trivial (0 ≤ *d* ≤ 0.19), small (0.20 ≤ *d* ≤ 0.49), moderate (0.50 ≤ *d* ≤ 0.79), or large (*d* ≥ 0.80) for the post-hoc analyses. The significance level was set *a priori* at *p* < .05 for all analyses.

## Results

**Fig. 2:**
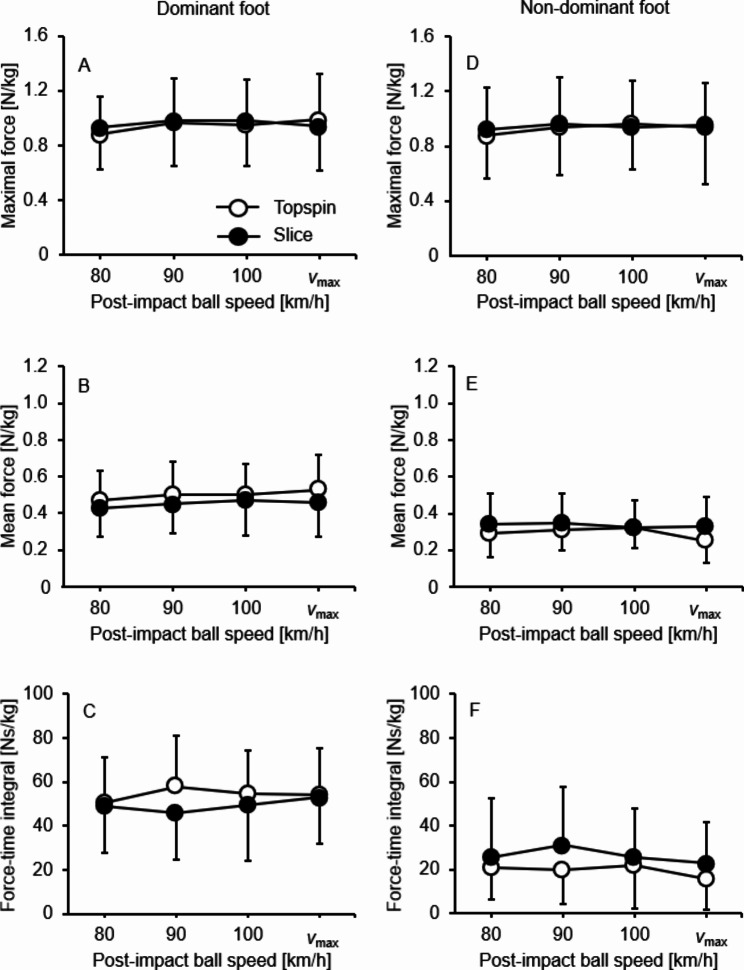
Plantar pressure values (mean and standard deviation) per post-impact ball speed level for the topspin (white circles) versus slice (black circles) longline forehand groundstrokes by foot dominance (i.e., dominant vs. non-dominant)

Descriptive (mean values and standard deviations) and inference (repeated measures ANOVA) statistics are shown in Fig. 2A–F; Table 1, respectively. The maximum post-impact ball speeds amounted to 137.1 ± 9.3 km/h (range: 120–160 km/h) for the topspin and 116.1 ± 9.6 km/h (range: 108–143 km/h) for the slice longline forehand groundstrokes.

**Table 1:** Inference statistics for the main and interaction effects

Outcome	Main effect: BS	Main effect: ST	Main effect: FD	Interaction effect BS × ST	Interaction effect BS × FD	Interaction effect ST × FD	Interaction effect BS × ST × FD
Maximal force [N/kg]	0.060 (0.16)	0.377 (0.06)	0.721 (0.01)	0.211 (0.10)	0.873 (0.02)	0.942 (0.01)	0.503 (0.05)
Mean force [N/kg]	0.002 (0.29)	0.676 (0.01)	< 0.001 (0.68)	0.284 (0.09)	0.001 (0.31)	0.061 (0.23)	0.064 (0.16)
Force-time integral [Ns/kg]	0.028 (0.19)	0.335 (0.07)	< 0.001 (0.71)	0.081 (0.15)	0.245 (0.09)	0.274 (0.09)	0.311 (0.08)

Values are expressed as p-value (*η*_p_^2^-value). BS = ball speed; FD = foot dominance; ST = stroke type

### Maximal force

Neither main nor interaction effects were detected for any outcome measure.

### Mean force

There were significant main effects of ball speed (*p* = .002, *η*_p_^2^ = 0.29) and foot dominance (*p* < .001, *η*_p_^2^ = 0.68). Further, the ball speed × foot dominance (*p* = .001, *η*_p_^2^ = 0.31) interactions reached the level of significance. Post-hoc tests revealed significantly larger values during topspin compared to slice groundstroke for the dominant (90 km/h: *p* = .016, *d* = 0.34; *v*_max_: *p* = .026, *d* = 0.36) but lower values for the non-dominant foot (80 km/h: *p* = .046, *d* = 0.31; 90 km/h: *p* = .050, *d* = 0.28; *v*_max_: *p* = .009, *d* = 0.58). For the topspin, GLM contrasts revealed significant increases in mean force for the dominant foot from 80 km/h to 90 km/h (*p* = .009, *η*_p_^2^ = 0.40), 100 km/h (*p* = .038, *η*_p_^2^ = 0.27), and *v*_max_ (*p* = .002, *η*_p_^2^ = 0.50) and for the non-dominant foot from 80 km/h to 90 km/h (*p* = .045, *η*_p_^2^ = 0.26). For the slice, GLM contrasts showed significant increases in mean force for the dominant foot from 80 km/h to 90 km/h (*p* = .045, *η*_p_^2^ = 0.26) but not for the non-dominant foot.

### Force-time integral

There were significant main effects of ball speed (*p* = .028, *η*_p_^2^ = 0.19) and stroke type (*p* < .001, *η*_p_^2^ = 0.71) but no significant interaction effects.

## Discussion

Partly in line with our first hypothesis stating that an increase in post-impact ball speed will result in plantar pressure changes in the dominant foot (i.e., increase) versus non-dominant foot (i.e., decrease) regardless of stroke type, we exclusively detected significant increases in mean forces in the dominant (topspin and slice) and non-dominant (topspin) foot. This finding contradicts those from our previous study [7], where we detected an increase in force values for the dominant foot but a decrease for the non-dominant foot when post-impact ball speed increased from 100 km/h to maximum while performing the longline topspin forehand (players were free to decide the stance style). One possible reason could be that in the present study stroke style (forehand), stroke direction (longline), and stance style (square) were identical for both stroke types. Additionally, in both stroke types, force generation from the legs occurs by means of weight shifting in the forward direction [4, 10], which most likely explains the increase in force in both the dominant and non-dominant leg.

Again, partly in accordance with our second hypothesis stating larger values during topspin compared to slice longline groundstrokes regardless of post-impact ball speed level, we observed significantly larger mean forces for the dominant foot but significantly lower values for the non-dominant foot. To our knowledge, this is the first study that applied kinetic analyses for the comparison of topspin and slice groundstrokes. Consequently, the results can only be interpreted in the light of studies that used other biomechanical analyses. A previous publication [1] applied high-speed cinematographic technique and found different racket trajectories between topspin and slice groundstrokes, which in turn require different leg drive. Specifically, during topspin stroke the racket head performs the forward swing at about hip height. To follow the racket movement, a forceful push-off from the dominant foot together with a forward-upward movement of the body is required [11]. Contrary, during slice stroke the outward movement is backward-upward, which means that the push-off from the dominant foot is lower [11]. Due to the subsequent forward-downward movement and the hitting point being close to the body [1], this results in a powerful push-off from the non-dominant leg [11].

From a practical perspective, the varying mean forces with respect to stroke type and foot dominance imply that specific physical exercises should be applied to obtain an optimal plantar pressure distribution for both factors. For example, bilateral exercises (i.e., leg press, dumbbell lunge, and squats) mainly involving the glutes and quadriceps should be performed to achieve increases in strength for the dominant and non-dominant foot. Additionally, unilateral exercises (i.e., single leg jumps, hops, and landings) predominately including the glutes, quadriceps, hamstrings, and calves should be applied in players that frequently use topspin strokes to improve strength in the dominant foot and to enhance the stabilising function of the non-dominant foot [12].

## Conclusion

We examined how plantar pressure data change when longline forehand topspin and slice groundstrokes were performed with increased post-impact ball speeds (i.e., 80 km/h, 90 km/h, 100 km/h, *v*_max_). Mean forces increased in the dominant (topspin and slice) and non-dominant (topspin) foot when post-impact ball speed was increased. Comparing topspin and slice groundstrokes revealed larger mean forces for the dominant foot but significantly lower values for the non-dominant foot. This indicates that specific physical exercises related to stroke type (topspin vs. slice) and foot (dominant vs. non-dominant) seem to be necessary to optimally distribute plantar pressure in tennis.

### Limitations

Firstly, we investigated only female tennis players, which limits the transfer of findings to male players. Secondly, players with a ITN ≤ 2 were studied. Thus, the results cannot be generalised to players with a lower skill level. Thirdly, the assessment was restricted to kinetic parameters, which does not allow statements about other biomechanical measures (e.g., kinematics of muscle activity). Lastly, the pressure-detecting insoles can only record the vertical force component and the horizontal component is not considered.

## Data Availability

The data generated and analysed during the present study are not publicly available due to ethical restrictions but are available from the corresponding author upon reasonable request.

## Abbreviations

ANOVA: Analysis of variance
BS: ball speed
FD: foot dominance
ITN: International Tennis Number
ST: stroke type

## References

[1] Elliott B, Marsh T (1989). A biomechanical comparison of the topspin and backspin forehand approach shots in tennis. J Sports Sci.

[2] Takahashi K, Elliott B, Noffal G (1996). The role of upper limb segment rotations in the development of spin in the tennis forehand. Aust J Sci Med Sport.

[3] Johnson CD, McHugh MP, Wood T, Kibler B (2006). Performance demands of professional male tennis players. BJSM.

[4] Knudson D (2006). Biomechanical principles of tennis technique: using science to improve your strokes.

[5] Genevois C, Reid M, Creveaux T, Rogowski I (2020). Kinematic differences in upper limb joints between flat and topspin forehand drives in competitive male tennis players. Sports Biomech.

[6] Shimokawa R, Nelson A, Zois J. Does ground-reaction force influence post-impact ball speed in the tennis forehand groundstroke? Sports Biomech. 2020;1–11. 10.1080/14763141.2019.1705884. 10.1080/14763141.2019.1705884 32026748

[7] Lambrich J, Muehlbauer T (2023). Plantar pressure is changed to increase post-impact ball speed during longline forehand and backhand groundstroke in elite female tennis players. Front Sports Act Living.

[8] Lambrich J, Muehlbauer T (2023). Biomechanical analyses of different serve and groundstroke techniques in tennis: a systematic scoping review. PLoS ONE.

[9] Lambrich J, Hagen M, Schwiertz G, Muehlbauer T (2023). Concurrent validity and test-retest reliability of pressure-detecting insoles for static and dynamic movements in healthy young adults. Sens (Basel).

[10] Kawamoto Y, Iino Y, Yoshioka S, Fukashiro S (2019). Directionally compensated mechanical work provided by the shoulder leads to similar racket velocities during open and square stance forehand groundstrokes in tennis. Eur J Sport Sci.

[11] German Tennis Federation. Tennis Curriculum - Tennis Theory; 2023.

[12] Lambrich J, Muehlbauer T (2022). Effects of athletic training on physical fitness and Stroke velocity in healthy youth and adult tennis players: a systematic review and meta-analysis. Front Sports Act Living.

